# Hepatocellular Carcinoma Around the Clock

**DOI:** 10.3390/curroncol33010032

**Published:** 2026-01-07

**Authors:** Mariana Verdelho Machado

**Affiliations:** 1 Clínica Universitária de Gastrenterologia, Faculdade de Medicina, Universidade de Lisboa, 1649-028 Lisbon, Portugal; mverdelhomachado@gmail.com; 2 Unidade de Oncologia Digestiva, Fundação Champalimaud, 1400-038 Lisbon, Portugal; 3 Serviço de Gastrenterologia, Hospital de Vila Franca de Xira, 2600-009 Vila Franca de Xira, Portugal

**Keywords:** hepatocellular carcinoma, circadian rhythm, chronotherapy

## Abstract

The dramatic shift in human behavior from hunter-gatherer to modern lifestyles has led to a systematic disruption of the human circadian cycle. Contributors include night-shift work, jet lag, and less intuitive but widespread factors, such as exposure to artificial light at night and irregular eating schedules. Circadian disruption is classified as a Group 2A carcinogen by the International Agency for Research on Cancer (IARC). Hepatocellular carcinoma (HCC) is the third most deadly cancer worldwide, with a rising prevalence in Western countries, largely driven by increasing rates of obesity and steatotic liver disease-associated hepatocarcinogenesis. Emerging evidence suggests that circadian disruption plays a significant role in HCC pathogenesis. Several genes involved in metabolism, cell survival, and immunosurveillance are under the control of circadian rhythms. Experimental preclinical data and epidemiological studies have indicated a strong association between circadian disruption and HCC development. Moreover, molecular signatures related to circadian regulation appear to accurately predict the prognosis of patients with HCC. The concept of chronotherapy is also gaining interest, with studies suggesting improved immunotherapy effectiveness when immune checkpoint inhibitors are administered in the morning. This review summarizes the current literature on the impact of circadian disruption on HCC pathogenesis, prognosis, and treatment.

## Simple Summary

Lifestyle-related circadian rhythm disruptions favor the development of hepatocellular carcinoma (HCC) both indirectly (by promoting precursor conditions such as metabolic dysfunction-associated liver disease and liver cirrhosis) and directly through intrinsic oncogenic effects. Population-level interventions that promote circadian realignment may help prevent the increasing incidence of HCC. Assessing circadian disruption could also help determine the prognosis of individual patients and their potential responses to therapy. Therapy strategies should incorporate chronotherapy principles, aiming not only to realign patient’s circadian cycles but also to optimize the timing of therapeutic delivery according to circadian variations in pharmacokinetics and pharmacodynamics to improve efficacy.

## 1. Introduction

Hepatocellular carcinoma (HCC) is the third leading cause of cancer-related deaths worldwide [1]. This mortality ranking contrasts with its position as the sixth most common cancer globally, reflecting the disease’s poor survival rates, with fewer than 20% of patients alive five years after diagnosis [2]. These statistics are even more concerning given predictions that, over the next 20 years, both the incidence and mortality of HCC will increase by approximately 50% [3]. The high mortality associated with HCC is due to both late diagnosis (with only 15–20% of patients being diagnosed at early, potentially curable stages) and the tumor’s aggressiveness and low treatment response rates [4,5]. The epidemiology of HCC etiology is also shifting: while chronic viral hepatitis is declining, steatotic liver disease (SLD), metabolic dysfunction-associated (MASLD) and alcohol-associated, has become the fastest growing cause of HCC [6]. As a result, lifestyle-related factors now account for approximately 52% of the attributable risk for HCC, including alcohol use, tobacco smoking, poor diet quality, and physical inactivity [7]. Lifestyle factors that disrupt circadian rhythms, such as abnormal sleep duration and night-shift work, may also significantly impact HCC epidemiology [8].

Indeed, “night-shift work”, and “shift work involving circadian disruption”, have been considered probable carcinogenics (Group 2A) by the International Agency for Research on Cancer (IARC) in 2007 and reaffirmed in 2019 [9].

The word circadian comes from the Latin *circa* (approximately) and *diem* (day). Most organisms, including animals and plants, exhibit circadian rhythms, reflecting their need to adapt to the environmental changes produced by the daily light–dark cycle generated by the Earth’s rotation around its axis [10]. Virtually all organs and cells display circadian rhythms that are hierarchically regulated by a central clock in the suprachiasmatic nuclei. Importantly, most physiological functions are under circadian control, including molecular mechanisms that, when disturbed, may contribute to cancer development, such as cell proliferation and differentiation, DNA damage repair, apoptosis, autophagy, anabolic and catabolic processes, and oxidative stress [11]. In the liver, approximately 15% of the transcriptome is regulated by circadian genes [12].

There is a significant amount of evidence from preclinical, epidemiological, and clinical research suggesting a strong association between circadian rhythm disruption and HCC development and poor prognosis. Furthermore, growing evidence suggests that chronotherapy may affect the treatment response to radiation and immunotherapy in patients with HCC.

This review summarizes the clinical evidence on the impact of circadian rhythms on the development, treatment, and prognosis of HCC and describes the potential mechanisms underlying these associations.

## 2. Physiology of the Circadian Rhythm

The maestro regulating the circadian rhythm is the central clock, located in the suprachiasmatic nuclei, which responds to external cues or zeitgebers, such as light, food intake, physical activity, and temperature [13]. Although peripheral clocks are under the control of the central clock via neuronal signals (mainly the autonomic nervous system), hormonal signals (e.g., cortisol and melatonin), and metabolic cues, they can also autonomously entrain circadian rhythms [14].

Rhythmicity is maintained by the oscillatory expression of several transcription factors, achieved through autoregulatory transcription loops [15]. At the top of this molecular hierarchy are two transcription factors that heterodimerize: circadian locomotor output cycle kaput (CLOCK) and brain and muscle ARNT-like-1 (BMAL1) [16]. At the start of the circadian day, the CLOCK-BMAL1 complex activates transcription by binding to E-box enhancer elements in the promoters of thousands of target genes [17], including period family genes (PER1, PER2, and PER3) [18], and cryptochrome family genes (CRY1 and CRY2) [19].

PER and CRY proteins heterodimerize and act as transcription repressors, inhibiting CLOCK-BMAL1 activity at night [20]. They are subsequently phosphorylated, PER proteins by casein kinase 1 (CK1) [21] and CRY proteins by other kinases such as AMPK [22], and ubiquitinated by E3 ligases, targeting them for proteasomal degradation [23]. This process restores CLOCK-BMAL1 activity, initiating a new cycle [20].

Other CLOCK-BMAL1 targets include retinoic acid-related orphan nuclear receptors (ROR) and REV-ERB. RORs act as transcriptional activators, whereas REV-ERBs act as repressors. Both compete for binding to RORE elements in the BMAL1 promoter, exerting opposing effects on BMAL1 expression [24].

**Figure 1:**
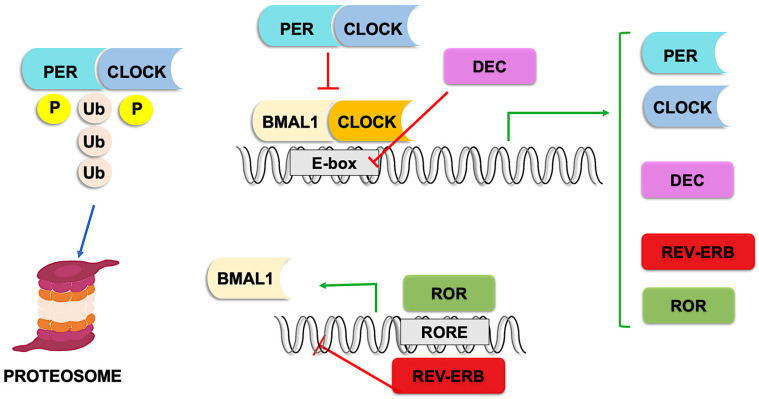
Molecular pathways of circadian clocks. The heterodimer BMAL1-CLOCK is a transcription factor for circadian-controlled genes including PER, CRY, DEC, REV-ERB, and ROR. Two autoregulatory feedback loops inhibits BMAL1-CLOCK action, either by direct inhibition (the heterodimer PER-CLOCK) or by competition with DNA binding (DEC). A third loop modulates BMAL1 expression, either promoting (ROR) or repressing it (REV-ERB). BMAL-1, brain and muscle ARNT-like-1; CLOCK, circadian locomotor output cycle kaput; PER, period; CYR, cryptochrome; DEC, differentiation embryo chondrocytes; RORs, retinoic acid-related orphan nuclear receptors. E-box and RORE cis regulatory elements. Green arrows indicate promotion of transcription and red ones inhibition.

Additionally, basic helix-loop-helix proteins called differentiated embryo chondrocytes (DECs) are also CLOCK-BMAL1 targets. In a negative feedback loop, DECs inhibit CLOCK-BMAL1 activity by competing for E-box binding [25] (Figure 1).

BMAL1 can alternatively heterodimerize with neuronal PAS domain protein-2 (NPAS2), broadening the range of target genes under circadian regulation [20].

Another key participant in circadian rhythmicity is TIMELESS, which interacts with PER and CRY proteins to promote their nuclear localization and stability, thereby enhancing their repression of CLOCK-BMAL1 activity [26,27].

Circadian regulation also occurs during post-translational modifications. For example, (1) MAPK-dependent phosphorylation of BMAL1 at threonine 534 induces a conformational change that reduces its ability to bind E-box elements, inhibiting its transcriptional activity [28]; (2) SUMOylation of BMAL1 at lysine 259 is circadian and enhances BMAL1 activity [29]; (3) Acetylation of BMAL1 by CLOCK also increases its activity [30], whereas SIRT1-dependent deacetylation decreases it [31].

## 3. Possible Mechanisms Linking Circadian Disruption and HCC

Up to 40% of the transcriptome is under circadian control [32], regulating multiple functions essential for maintaining homeostasis (such as metabolism, immune responses, cell cycle progression, and cell death), which, when disrupted, may promote cancer.

Mouse models harboring mutant circadian genes exhibit an increased risk of cancer development. In these models, HCC is the second most frequent cancer, following lymphomas [33].

Mouse models exposed to chronic jet leg develop persistent metabolic derangements that lead to steatohepatitis and spontaneous HCC [34,35]. A central mechanism driving hepatic steatogenesis, fibrogenesis, and carcinogenesis in this model was the activation of the nuclear receptor constitutive androstane receptor (CAR), mediated by sympathetic dysfunction and cholestasis. Chronic jet leg disrupts the synchronization between the central and peripheral clocks via the sympathetic nervous system. During the sleep phase, sympathetic tone increases, inducing beta-adrenergic signaling, which activates protein kinase A (PKA)-dependent CAR signaling and MAPK-dependent AP-1 activation. AP-1 then upregulates c-Myc, promoting cell growth and survival [33]. Chronic jet leg also induces genome-wide metabolic reprogramming, altering cholesterol and bile acids metabolism, and ultimately causing intrahepatic cholestasis. Elevated bile acids levels further activate CAR. CAR is a potent driver of carcinogenesis through survival and proliferation pathways, including Wnt/beta-catenin signaling [34], which is one of the earliest and most important genetic alterations in human HCC [36]. Wnt/beta-catenin signaling is associated with HCC stemness, tumor growth, metastasis, and therapy resistance [36].

Another key pathway in HCC development is the Hedgehog pathway [37]. Hedgehog signaling in hepatocytes is under circadian control and, in turn, exerts negative feedback on the circadian clock by promoting the expression of the repressors PERs [38]. Experimental BMAL1 knockdown increases Hedgehog pathway activation. Notably, most HCC exhibit Hedgehog activation, which correlates with tumor growth, invasiveness, metastasis, and treatment resistance [39].

Circadian disruption can also blunt PER1-mediated activation of ATM and alter CLOCK-BMAL1-dependent SIRT1-mediated acetylation of p53. Loss of ATM and p53 activity results in defective apoptosis, impaired cell cycle arrest, and reduced DNA repair, leading to increased tumor growth and genomic instability [40].

The PI3K/AKT/mTOR signaling pathway is also under circadian regulation [41], which in turn modulates circadian clock function, including through CLOCK serine 845 phosphorylation, which decreases its nuclear localization [41] and through mechanisms required for CLOCK-BMAL1 heterodimerization and DNA binding [42]. This pathway plays a central oncogenic role in HCC, driving cell proliferation and survival and inducing metabolic reprogramming toward the Warburg effect (i.e., glycolysis to lactate production even in the presence of oxygen) [43]. In addition, this pathway promotes epithelial–mesenchymal transition and upregulates matrix metalloproteinases, thereby enhancing invasiveness and metastasis [44,45].

Upregulation of NPAS2, an alternative heterodimer to the BMAL1 partner, in some HCC tumors is associated with enhanced expression of CDC25A, which dephosphorylates cyclin-dependent kinases, promoting cell proliferation and inhibiting mitochondria-dependent intrinsic apoptosis. NPAS2 upregulation is associated with worse recurrence-free and overall survival [46].

**Figure 2:**
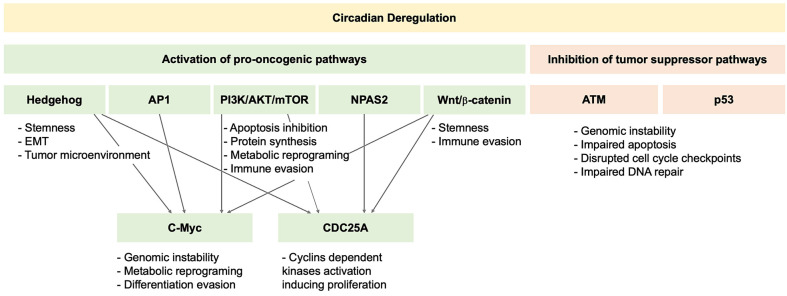
Molecular pathways interlinked between circadian rhythms and hepatocarcinogenesis.

In conclusion, the circadian pathway intersects with several carcinogenic pathways that play key roles in hepatocarcinogenesis. Indeed, HCC tumors presenting molecular signatures of circadian dysfunction activate pro-carcinogenic pathways, including Wnt/β-catenin, Hedgehog, and PI3K/ATK/mTOR, and inhibit tumor suppressors such as ATM and p53 [36,47,48] (Figure 2).

## 4. Epidemiological Evidence Linking Circadian Disruption and HCC

Circadian misalignment seems to be associated with an increased risk of HCC, although some conflicting reports highlight the need for further research on this topic. Population-level epidemiological studies evaluating HCC incidence across geographical longitudes within the same time zone have consistently shown an increased HCC risk from east to west [49,50,51], after adjustment for confounding factors such as the prevalence of health conditions (including obesity and diabetes mellitus), lifestyle, shift work occupation, socioeconomic status, and environmental factors. Each degree of longitude westward corresponds to a 4 min delay in sunrise [49]. Cities located on the western edges of a time zone may therefore experience more than 60 min of circadian misalignment compared with those on the eastern edge, resulting in a phase delay characterized by reduced morning light exposure and increased light exposure later in the day [52]. In contrast, a prospective cohort study including nearly half a million individuals did not observe an association between residential outdoor light-at-night exposure and HCC risk [53]. However, outdoor light-at-night exposure is difficult to quantify accurately, which may have led to an underestimation of its effect. Furthermore, although meta-analyses support an association between night-shift work and increased cancer-related mortality [54], the largest epidemiological study specifically assessing HCC unexpectedly reported a decreased liver cancer risk among Japanese men engaging in rotating shift work [55]. Of note, the Japanese study did not account for variations in shift work patterns, duration, intensity, or underlying HCC etiology. Moreover, the cohort—aged 40–79—may reflect a healthy-worker effect, as older individuals are less likely to engage in night shifts and those who remain cancer-free by age 79 are unlikely to experience substantial additional risk from continued exposure.

Sleep patterns appear to modulate the risk of HCC. Similar to what has been described for MASLD, cardiovascular diseases, and overall mortality [56], there seems to be a U-shaped association between sleep duration and HCC risk, with a nadir between 6 and 8 h of sleep [57,58], after adjustment for socioeconomic status, lifestyle habits, body mass index, and comorbidities, including diabetes mellitus and hypertension. Large epidemiological studies suggest that sleeping 5 h or less per day increases the risk of HCC by approximately 25%, whereas sleeping 9 h or more increases the risk by 25% to up to 2-fold (and up to 3-fold in obese patients) [57,58,59]. Daytime napping, particularly for more than one hour, which disrupts the circadian cycle, has also been associated with an almost 50% increased risk of HCC [58]. In addition to sleep quantity, sleep quality also appears to play an important role, with large epidemiological studies suggesting an almost 50% increase in HCC risk among patients with poor sleep quality [8,60]. It has further been estimated that nearly 15% of liver cancers could be prevented if the population maintained healthy sleep patterns [60]. It is important to note that epidemiological studies may be affected by reverse causality. In particular, some liver diseases, including liver cirrhosis, a premalignant condition that can progress to HCC, have been linked to sleep disorders, such as increased sleep fragmentation, reduced sleep efficiency, and longer daytime naps [56]. However, several design features mitigated the risk of reverse causation: all studies employed prospective cohort designs; three excluded participants with prior history of malignancy [8,58,59]; three excluded those with pre-existing chronic liver disease [57,59,60], with one additionally excluding individuals with hepatitis B or C infection [58]; and two excluded cases occurring within the first 2–5 years of follow up [58,60]. A summary of the epidemiological studies is presented in Table 1.

Human HCC frequently exhibits disturbances in circadian gene expression. Studies comparing gene expression in HCC tumors and adjacent non-tumor tissues have consistently shown decreased expression of several circadian genes in tumors, including PER1-3, CYR2, TIM, and NPAS2 [46,61,62,63,64,65,66,67,68]. In addition to reduced expression, some core clock components, such as PER1 and CRY1, lose their circadian oscillation in HCC samples [69]. Interestingly, these changes in gene expression are rarely associated with gene mutations [66]; instead, epigenetic modifications are the primary drivers of these dysregulations. For example, promoter methylation often silences these genes [61]. In HCC tumors, histone methyltransferases, such as EZH2, are overexpressed [61]. EZH2 itself exhibits circadian oscillation in expression [70] and can epigenetically modify clock genes, including members of the PER and CRY families [71]. Another epigenetic mechanism in HCC involves the overexpression of histone deacetylases (HDACs) [72,73], which promote chromatin condensation and reduce the accessibility of the transcriptional machinery to the promoters of core clock genes, such as BMAL1 and members of the PER and CRY families [74,75]. Non-coding RNAs may also contribute to circadian gene dysregulation in HCC. For instance, the expression of miR-195-5p and miR-122-5p is decreased in HCC samples, which appears to indirectly reduce the expression of circadian genes [76,77]. Conversely, the long non-coding RNA HULC is upregulated in HCC and increases CLOCK expression while altering its periodicity through complementary base pairing with the untranslated region of CLOCK mRNA [78].

These diverse mechanisms of circadian gene dysregulation underscore their fundamental role in HCC pathology, with significant prognostic implications. Different gene signatures, reflecting combinations of differentially expressed circadian genes in tumor and non-tumor tissues, have shown good accuracy in predicting prognosis. High-risk signatures, characterized by greater disruption of circadian rhythms, are associated with aggressive malignant highly proliferative phenotypes, higher pathological grade, advanced staging, and poorer overall survival [47,48,67,79,80,81,82,83,84]. They are also correlated with elevated expression of immune checkpoint genes, increased immune infiltration, a less immunocompetent phenotype, and reduced responsiveness to immunotherapy [67,82,84].

In addition to altered gene expression, germinal genetic variants of clock genes are critical factors linked to the prognosis of HCC patients. For instance, single nucleotide polymorphisms (SNPs) in PER1, PER3, CRY1, and NPAS2 have been associated with recurrence-free and overall survival following surgical resection [85] and transarterial chemoembolization [86,87]. A dose-dependent effect was observed, with a higher number of variants corresponding to a stronger impact on prognosis.

## 5. Chronotherapy for HCC

The term *chronotherapy* encompasses two distinct concepts: (1) interventions that align the circadian rhythm and (2) the scheduling of therapeutic interventions at specific times of day [52,88].

One way to entrain the circadian rhythm is by manipulating feeding schedules. In mouse models of HCC, time-restricting eating (TRE) reduces both the development and growth of liver tumors [89,90] and is associated with higher tumor differentiation and lower metastatic potential [91]. The antitumor effects of TRE may stem from circadian resynchronization, which occurs through several mechanisms: (1) fasting decreases CRY1 and PER proteins stability via AMPK-dependent pathways, enabling the restoration of clock genes oscillation [22,92]; (2) fasting increases SIRT1 activity, which interacts with CLOCK-BMAL1 and suppresses PER2 transcription [93]; and (3) feeding induces CRY gene expression in an mTOR-dependent but CLOCK-BMAL1 independent manner [94]. In addition, prolonged fasting may exert antitumor effects, as tumor cells are more vulnerable to nutrient depletion. Their higher susceptibility to fasting arises from a shift toward less energetically efficient anaerobic glucose metabolism, inability to downregulate cell proliferation and energy demands, and lack of compensatory autophagy under nutrient-depleted conditions [90,95]. Forcing tumor cells to shift from anaerobic metabolism to oxidative phosphorylation increases oxidative stress, DNA damage, and apoptosis in cells that maintain a high proliferative index [90]. Animal models have also shown that intermittent fasting, but not short-term fasting, modifies the tumor microenvironment by altering the extracellular matrix, vascularization, and increasing immune cell infiltration. These changes enhance antitumor drug delivery and affect cancer cell survival, invasion, and metastasis [90]. Preclinical in vitro and in vivo studies have suggested that fasting may increase the response to sorafenib treatment. One explanation could be sorafenib’s inhibition of mitochondrial respiration, which increases cellular vulnerability to nutrient depletion [95]. No clinical trials have yet evaluated TRE in human HCC, and caution is warranted regarding the potential risks of TRE in cirrhotic patients with HCC, as fasting increases the risk of sarcopenia in patients with cirrhosis [96].

Melatonin is produced in the pineal gland in coordination with the light–dark circadian cycle and conveys this information to regulate sleep time [97]. In addition to its chronobiological role, melatonin possesses antioxidant, anti-inflammatory, and antiproliferative properties [98,99], which contribute to its intrinsic antitumor activity in various cancers, including HCC [97]. In vitro studies using human HCC cell lines have shown that melatonin induces apoptosis and inhibits cell proliferation, epithelial–mesenchymal transition, migration, and invasiveness [100]. Melatonin also exhibits anti-angiogenic properties [101]. These effects are mediated through multiple mechanisms, including FOXA-2- and mTORC1-dependent inhibition of hypoxia-inducible factor-1 (HIF-1) [97,102], modulation of cAMP, ERK/MAPK-JNK, and MAPK-P38 signaling [103,104], NF-kB inhibition-dependent upregulation of tissue inhibitor of metalloproteinases-1 (TIMP-1) [105], and metabolic reprograming that counteracts the Warburg effect, by which cancer cells ferment glucose to lactate despite adequate oxygen availability [106]. In vivo HCC models have similarly demonstrated that melatonin administration abrogates tumor growth [97]. Notably, similar tumor growth inhibition was observed when animals were exposed to light-emitting diode illumination, which amplified nocturnal melatonin levels compared to cool white fluorescent lighting [106]. Melatonin treatment ultimately improved survival in these HCC models [107]. In vitro data also suggest that melatonin increases HCC cell sensitivity to sorafenib treatment [102,108]. It might even modulate immunotherapy responses, as exosomes from melatonin-treated HCC cells promote a more immunocompetent phenotype and suppress PD-L1 expression in vitro and in vivo [104,109]. A comprehensive analysis of 12 melatoninergic genes showed that tumor tissues exhibit reduced expression of nuclear and cytoplasmic melatonin receptor genes, as well as metabolic melatonin genes, compared to adjacent non-tumor tissues [110]. Lower tumor expression of melatonin-synthesizing enzymes has also been linked to increased mortality in patients with HCC [111]. Furthermore, among patients with HCC undergoing liver transplantation, lower pre-transplant melatonin levels were associated with a higher risk of 1-year post-transplantation mortality [112]. Despite the growing evidence supporting the rationale for melatonin as a therapeutic strategy for HCC, no clinical trials have evaluated melatonin treatment in human HCC.

Regarding the optimization of treatment timing, a seminal paper published nearly 30 years ago on colorectal cancer first reported that timed infusions of chemotherapy were associated with an increased response rate and reduced toxicity, allowing the administration of higher doses of fluorouracil [113]. Since then, several studies on different cancers have evaluated the effect of the timing of chemotherapy administration, with the majority describing decreased toxicity and some describing increased efficacy [114]. Prespecified time-of-day treatment delivery relies on circadian clock effects in different aspects of drug delivery, including absorption (by modulating gastric emptying, gut motility and blood flow, drug transporter and exporter expression [115,116,117]), distribution (by differences in cardiac output and blood flow across the day [118]), metabolism (for example, clock genes regulate the expression of cytochrome P450 enzymes [119,120,121,122]), and excretion [52].

HCC is insensitive to chemotherapy; however, in vitro and in vivo preclinical models suggest that the timing of the administration of the tyrosine kinase inhibitor sorafenib has an impact on tumor progression [123]. Data on the time-of-day administration of tyrosine kinase inhibitors in human HCC are still lacking.

HCC preclinical models have shown a therapeutic advantage and lower toxicity when radiation therapy was administered during the early activity phase or late inactivity phase, which corresponded to peaks of HCC cell proliferation [124,125]. In addition, adjusting radiation timing to circadian fluctuations in DNA repair machinery may impact treatment-related toxicity [88]. The effect of radiation timing on human HCC remains undetermined.

In the last five years, the establishment of immunotherapy for HCC treatment has revolutionized systemic therapy. Tumor cells can evade immune attack by expressing programmed death-ligand-1 (PD-L1), which binds to its corresponding receptor on immune cells, programmed cell death protein-1 (PD-1), thereby inhibiting immune cell activity. Additionally, cytotoxic T-lymphocyte antigen-4 (CTLA-4), which is expressed on T cells, suppresses T-cell responses by competing with the costimulatory receptor CD28, which is required for complete T-cell activation. Immune checkpoint inhibitors (ICI) are monoclonal antibodies that block PD-L1, PD-1, or CTLA-4, preventing their inhibitory effects and eliciting an antitumor immune response [126]. Some patients achieve long-term survival [127] but fewer than one-third experience an objective response [128]. Chronotherapy is one strategy to improve response rates. Retrospective studies on several metastatic cancers have suggested that early day administration of immunotherapy is associated with increased response rates and improved overall survival [126,129]. In HCC, preclinical models suggested that circadian-timed administration of immunotherapy, compared with continuous infusion, led to a 4-fold increase in the objective response rate and eliminated mortality [130]. More recently, a retrospective analysis of human HCC, including a total of 751 patients, reported that morning infusions of ICIs, compared with afternoon infusions, were associated with improved progression-free and overall survival and a higher objective response rate (45% vs. 33%) [131]. Despite providing valuable insights, this study has several limitations inherent to its retrospective design. First, unmeasured confounding factors may have influenced the observed associations, including variables such as liver function fluctuations (even though the study only included patients from Child-Pugh-Turcotte class A at baseline), comorbidities, prior therapies, tumor molecular profile, and adherence to treatment protocols. Second, selection bias cannot be entirely excluded, as patients included in retrospective cohorts may differ systematically from those not captured in the dataset. Third, timing and accuracy of clinical and laboratory measurements may vary, potentially affecting the assessment of predictors and outcomes. These limitations underscore the need for well-designed prospective studies to rigorously evaluate predictors of immunotherapy response in hepatocellular carcinoma. Although such trials are currently ongoing for melanoma (NCT07155317) and lung cancer (NCT05549037), comparable studies specifically addressing HCC are still lacking. The effects of immunotherapy timing on pharmacokinetics would be expected to manifest primarily before the drug reaches steady-state blood concentrations, which occurs between the second and fourth months of therapy [132], and hence the timing of the first infusions would be particularly relevant [126]. However, blood levels do not accurately reflect antibody levels within the tumor [133], as uptake is influenced by competition between tumor-infiltrating immune cells and lymphoid organs, which may exhibit circadian oscillations [134]. The immune response appears to be subject to circadian regulation. The number and function of tumor-infiltrating T cells vary throughout the day [135]. Several mechanisms have been proposed, including the regulation of T cell infiltration by endothelial circadian clocks, which drive oscillations in endothelial cell adhesion molecule expression [136]. The phenotype of tumor-associated immune cells is also under circadian control, with oscillating genes enriched in pathways related to metabolism, cell adhesion, differentiation, and T cell activation [137]. Notably, PD-1 expression in tumor-infiltrating T cells also follows a circadian pattern [135].

## 6. Conclusions

HCC is a deadly cancer with a rising incidence, particularly in Western countries, largely driven by steatotic liver diseases. Notably, more than 50% of the attributable risk is related to lifestyle factors, meaning that up to half of all HCC cases can be prevented. Modern lifestyles increasingly disregard circadian rhythms, with artificial light altering natural light–dark cycles and food being available around the clock. Disruption of the circadian cycle not only contributes to liver diseases that can progress to HCC but also appears to have intrinsic hepato-oncogenic effects.

The most effective way to reduce HCC mortality is by reducing its incidence. Strong educational efforts and government policies should be implemented to promote lifestyles that align with healthy circadian rhythms.

Systemic treatment of HCC has dramatically changed in the last five years with the advent of immunotherapy. However, only approximately one-third of patients respond to these treatments. Chronotherapy is gaining attention and appears to be a promising strategy for improving therapeutic responses. Assessing tumor circadian disruption may help identify patients who are most likely to benefit from the treatment.

## Figures and Tables

**Table 1:** Epidemiological studies on the relationship between circadian deregulation and HCC.

Study	Study Design	Factors Adjusted	Main Results
Geographical longitude and HCC incidence			
Gu, F. 2017 [50]	SEER data (2000–2012) from 607 counties across 11 U.S. states. Incidence rates for 23 cancers types.	Age	Per 5° westward increase in longitude within a time zone: HCC RR 1.11 [95%CI 1.05–1.18]
VoPham, T. 2018 [49]	SEER data (2000–2014) from 16 population-based US cancer registries. 56,347 HCC cases included.	Demographics Socioeconomic status Obesity, T2DM rates Lifestyle habits Shift work occupation Environmental factors	Per 5° westward increase in longitude within a time zone: HCC IRR 1.07 [95%CI 1.01–1.14]
Niu, J. 2024 [51]	State Cancer Profiles (2016–2020) from 2853 counties across 48 U.S. states. Incidence rates for 8 cancers types.	Demographics Socioeconomic status Obesity rate Altitude Environmental factors	Decreasing incidence of liver and bile duct cancers from west to east within a time zone
Residential outdoor LAN exposure (measured from satellite imagery) and HCC risk			
Park, Y. 2022 [53]	U.S. prospective cohort of 451,945 participants aged 50–71 years from the NIH-AARP Diet and Health Study (1995–1996). Median follow up 12.2 years.	Demographics Socioeconomic status BMI Lifestyle habits	No association between outdoor LAN exposure and HCC risk: RR 0.96 [95%CI 0.77–1.20], quartile 5 vs. 1
Night shift work and HCC risk			
Arafa, A. 2021 [55]	Japanese prospective cohort of 45,390 workers aged 40–79 years from the JACC Study (1988–1990). Median follow up 14.2 years.	Demographics Socioeconomic status Lifestyle habits	Rotation shift-work associates with increased HCC risk: HR 0.54 [95%CI 0.30–0.98]
Sleep patterns and HCC risk			
Hu, L.Y. 2013 [8]	Taiwan prospective cohort of 63,381 newly diagnosed sleep disorder patients from the Taiwan National Health Insurance Research Database (1996–2010). Median follow up 6.2 years.	Demographics	SIR for liver cancer 1.44 [95%CI 1.28–1.61]
Royse, K.E. 2017 [59]	U.S. prospective cohort of 139,368 postmenopausal women from the Prospective Women’s Health Institute Study (1993–1998). Median follow up 13.8 years.	Demographics Socioeconomic status Lifestyle habits BMI and T2DM	HCC HR compared with 6–8 H sleep: ≤5 H: 1.10 [95%CI 0.63–1.94] 6 H: 0.90 [95%CI 0.61–1.32] ≥9 H: 1.88 [95%CI 1.02–3.45]
Long, L. 2023 [58]	U.S. prospective cohort of 295,837 participants from the NIH-AARP Diet and Health Study (1995–1996). Median follow up 15.5 years.	Demographics Socioeconomic status Lifestyle habits	HCC HR compared with 7–8 H sleep: <5 H: 2.00 [95%CI 1.22–3.26] 5–6 H: 1.15 [95%CI 0.91–1.45] ≥9 H: 1.63 [95%CI 1.04–2.65] Napping > 1 H: 1.46 [95%CI 1.04–2.06]
Wang, W. 2024 [60]	UK prospective cohort of 408,560 participants free of CLD, aged 40–70 years from the UK Biobank (2006–2010). Median follow up 12.5 years.	Demographics Lifestyle habits BMI, lipid profile, HTN and T2DM	Healthy sleep score associates with decreased HCC risk: HR 0.53 [95%CI 0.37–0.77]
Wang, Q. 2024 [57]	UK prospective cohort of 489,261 participants aged 37–73 years from the UK Biobank (2006–2010). Median follow up 13.8 years.	Demographics Socioeconomic status BMI, T2DM, HTN Lifestyle habits	HCC HR compared with 7 H sleep: ≤5 H: 1.26 [95%CI 0.98–1.60] 6 H: 1.02 [95%CI 0.86–1.24] 8 H: 1.04 [95%CI 0.89–1.20] ≥9 H: 1.23 [95%CI 1.01–1.51]

BMI, body mass index; CI, confidence interval; CLD, chronic liver disease; HCC, hepatocellular carcinoma; HR, hazard ratio; HTN, hypertension; IRR, incidence rate ratio; JACC, Japanese Collaborative Cohort; LAN, light-at-night; NIH-AARP, National Institute of Health—American Association of Retired Persons; RR, relative risk; SEER, Surveillance, Epidemiology, and End Results; SIR, standardized incidence ratio; T2DM, type 2 diabetes mellitus.

## Data Availability

No new data were created or analyzed in this study.

## Abbreviations

The following abbreviations are used in this manuscript:

BMAL1	Brain and Muscle ARNT-Like-1
CAR	Constitutive Androstane Receptor
CLOCK	Circadian Locomotor Output Cycle Kaput
CRY	Cryptochrome
CTLA-4	Cytotoxic T-Lymphocyte Antigen-4
DEC	Differentiated Embryo Chondrocytes
HCC	HepatoCellular Carcinoma
HDAC	Histone DdeACetylase
IARC	International Agency for Research on Cancer
ICI	Immune Checkpoint Inhibitors
MARCKSL1	Myristoylated Alanine-Rich C Kinase Substrate-Like 1
MASLD	Metabolic dysfunction-Associated Steatotic Liver Disease
NNMT	Nicotinamide N-MethylTransferase
NPAS2	Neuronal PAS domain Protein-2
PD-1	Programmed cell Death protein-1
PD-L1	Programmed Death-Ligand-1
PER	Period
PKA	Protein Kinase A
ROR	Retinoic acid-related Orphan nuclear Receptors
SLD	Steatotic Liver Disease
SNP	Single Nucleotide Polymorphisms
TIMP-1	Tissue Inhibitor of MetalloProteinases-1
TRE	Time Restricting Eating
